# Supplementary material for: High-throughput metabarcoding reveals the effect of physicochemical soil properties on soil and litter biodiversity and community turnover across Amazonia
Source: PeerJ. 2018 Sep 25;6:e5661. doi: 10.7717/peerj.5661 (PMC6161700; doi:10.7717/peerj.5661)
Supplement: Supplemental Information 2 [file peerj-06-5661-s002.docx]

**SUPPLEMENTARY MATERIAL FOR:**

**High-throughput metabarcoding reveals the effect of physicochemical soil properties on soil and litter biodiversity and community turnover across Amazonia**

Camila D. Ritter^1,2*^, Alexander Zizka^1,2^, Fabian Roger^3^, Hanna Tuomisto^4^, Christopher Barnes^5^, R. Henrik Nilsson^1,2^, Alexandre Antonelli^1,2,6,7^

^1^ Gothenburg Global Biodiversity Centre, Box 461, SE-405 30 Göteborg, Sweden

^2^ Department of Biological and Environmental Sciences, University of Gothenburg, Box 463, 405 30 Göteborg, Sweden

^3^ Centre for Environmental and Climate Research, Lund University, Sölvegatan 37, SE-223 62 Lund, Sweden

^4^ Department of Biology, University of Turku, Turku, Finland

^5^ Natural History Museum of Denmark, University of Copenhagen, Solvgade 83, DK-1307 Copenhagen, Denmark

^6^ Gothenburg Botanical Garden, Carl Skottsbergs gata 22A, SE-41319 Göteborg, Sweden

^7^ Department of Organismic and Evolutionary Biology, Harvard University, 26 Oxford St., Cambridge, MA 02138 USA

*Corresponding author: Camila Ritter, camila.ritter@bioenv.gu.se. Phone: +46 723924447, Carl Skottsbergs gata 22B - P.O. Box 461 - SE 405 30 - Göteborg – Sweden

Contents

[Table S1 3](#_Toc521324799)

[Table S2 5](#_Toc521324800)

[Table S3 6](#_Toc521324801)

[Table S4 7](#_Toc521324802)

[List of figures: 8](#_Toc521324803)

[Figure S1 8](#_Toc521324804)

[Figure S3: 10](#_Toc521324805)

Table S1**:** Mean of operational taxonomic units (OTUs) rarefied to the lowest number of reads obtained from a single plot (22,209 for 16S and 25,144 for 18S; Fig S2) and the effective number of OTUs derived from the unrarefied read counts. The samples for which soil variables were directly measured are marked with “YES”, and the others sport values inferred by calculation of the regression weights from the observed data.

| **ID** | **OTUs mean soil 16S rarefied** | **Effective number of OTU soil (16S)** | **OTUs mean litter 16S rarefied** | **Effective number of OTU litter (16S)** | **OTUs mean soil 18S rarefied** | **Effective number of OTU soil (18S)** | **OTUs mean litter 18S rarefied** | **Effective number of OTU litter (18S)** | **Soil data** |
| --- | --- | --- | --- | --- | --- | --- | --- | --- | --- |
| BCIGP1 | 1378.11 | 262.17 | 1250.87 | 142.01 | 271.58 | 96.63 | 160.41 | 8.10 | Yes |
| BCIGP2 | 1432.34 | 319.49 | 1512.63 | 373.40 | 208.74 | 40.35 | 331.40 | 115.54 | Yes |
| BCIGP3 | 1553.50 | 369.64 | 1343.76 | 126.64 | 281.82 | 65.65 | 331.25 | 118.65 | Yes |
| BCTFP1 | 1412.69 | 286.43 | 1441.08 | 342.68 | 235.31 | 61.85 | 238.46 | 66.66 | Yes |
| BCTFP2 | 1261.97 | 223.33 | 1373.28 | 324.13 | 224.98 | 52.23 | 272.45 | 85.07 | Yes |
| BCTFP3 | 1203.32 | 197.12 | 1319.84 | 295.57 | 201.18 | 39.56 | 198.86 | 50.51 | Yes |
| BCVZP1 | 1346.84 | 226.04 | 1334.09 | 199.96 | 159.04 | 16.15 | 327.73 | 97.93 | Yes |
| BCVZP2 | 1460.06 | 267.01 | 1402.10 | 224.51 | 184.70 | 23.29 | 177.72 | 31.27 | Yes |
| BCVZP3 | 1414.13 | 267.40 | 1201.57 | 159.64 | 194.29 | 26.47 | 156.64 | 21.07 | Yes |
| CUICAMP1 | 1248.07 | 180.31 | 1218.09 | 242.37 | 338.24 | 150.85 | 285.84 | 119.59 | Yes |
| CUICAMP2 | 905.33 | 262.04 | 993.41 | 189.78 | 263.64 | 119.29 | 215.77 | 72.99 | Yes |
| CUICAMP3 | 1128.76 | 133.02 | 1180.12 | 152.63 | 287.26 | 108.08 | 220.00 | 63.28 | Yes |
| CUIIGP1 | 953.91 | 125.44 | 984.34 | 175.98 | 109.86 | 8.00 | 206.82 | 46.00 | Yes |
| CUIIGP2 | 907.44 | 122.25 | 807.74 | 119.15 | 93.87 | 10.12 | 126.58 | 22.11 | Yes |
| CUIIGP3 | 850.54 | 90.55 | 831.53 | 127.36 | 101.10 | 10.79 | 150.95 | 33.03 | Yes |
| CUITFP1 | 1027.64 | 170.00 | 739.20 | 85.82 | 257.28 | 80.88 | 87.76 | 5.43 | Yes |
| CUITFP2 | 1058.81 | 167.88 | 1198.27 | 241.65 | 259.05 | 88.01 | 358.07 | 216.61 | Yes |
| CUITFP3 | 864.87 | 129.46 | 857.29 | 226.92 | 231.17 | 81.58 | 283.73 | 145.82 | Yes |
| CXNCAMP1 | 1099.17 | 176.68 | 1567.59 | 386.95 | 273.97 | 79.19 | 167.98 | 23.07 | Yes |
| CXNCAMP2 | 1009.96 | 157.23 | 1306.90 | 273.18 | 245.73 | 62.45 | 242.77 | 76.30 | Yes |
| CXNCAMP3 | 479.02 | 102.65 | 1362.30 | 300.80 | 133.89 | 26.74 | 151.36 | 19.23 | Yes |
| CXNIGP1 | 1027.89 | 131.58 | 929.17 | 66.76 | 227.97 | 62.56 | 150.02 | 27.59 | Yes |
| CXNIGP2 | 1094.46 | 123.91 | 944.58 | 61.43 | 258.60 | 76.58 | 125.38 | 18.19 | Yes |
| CXNIGP3 | 931.42 | 139.66 | 1003.13 | 140.55 | 236.72 | 61.45 | 138.59 | 25.17 | No |
| CXNTFP1 | 1059.29 | 150.80 | 1261.51 | 299.54 | 389.12 | 243.93 | 100.48 | 9.74 | Yes |
| CXNTFP2 | 1085.45 | 174.21 | 1298.92 | 279.55 | 235.97 | 81.60 | 108.02 | 13.63 | Yes |
| CXNTFP3 | 1180.06 | 200.04 | 1347.34 | 339.32 | 233.53 | 43.73 | 169.29 | 23.39 | Yes |
| CXNVZP1 | 1142.12 | 153.49 | 1003.86 | 59.98 | 99.76 | 9.15 | 114.53 | 16.07 | No |
| CXNVZP2 | 1221.42 | 132.84 | 1301.70 | 173.14 | 60.89 | 3.16 | 322.78 | 154.78 | Yes |
| CXNVZP3 | 1325.00 | 294.04 | 1241.78 | 166.67 | 304.50 | 78.79 | 150.18 | 20.92 | Yes |
| JAUCAMP1 | 1318.89 | 176.14 | 1309.23 | 182.19 | 223.18 | 34.44 | 169.51 | 32.72 | Yes |
| JAUCAMP2 | 1530.34 | 381.12 | 1280.47 | 282.90 | 360.94 | 181.54 | 185.98 | 39.76 | Yes |
| JAUCAMP3 | 1296.99 | 257.75 | 1149.58 | 260.74 | 200.55 | 40.46 | 192.28 | 65.73 | No |
| JAUIGP1 | 1034.06 | 139.04 | 941.49 | 161.99 | 280.98 | 112.95 | 248.33 | 63.76 | Yes |
| JAUIGP2 | 1050.84 | 137.17 | 881.99 | 106.12 | 139.11 | 13.55 | 124.00 | 15.22 | Yes |
| JAUIGP3 | 956.62 | 128.97 | 924.15 | 130.06 | 266.28 | 96.14 | 282.88 | 106.72 | Yes |
| JAUTFP1 | 1021.85 | 144.28 | 847.43 | 154.45 | 335.00 | 126.94 | 103.17 | 7.06 | Yes |
| JAUTFP2 | 1130.92 | 186.57 | 895.62 | 173.51 | 250.45 | 63.48 | 171.97 | 23.11 | Yes |
| JAUTFP3 | 1016.35 | 93.96 | 631.77 | 78.14 | 275.28 | 60.46 | 63.09 | 8.76 | Yes |

Table S2: **Principal component analysis (PCA)** of the physical proprieties: silt, clay, and fine, coarse, and total sand fraction. Each principal component (PC) represents a proportion of variation of each variable. The table shows the total variance represented in each PC and the cumulative proportion of variance.

| **Variables** | **PC1** | **PC2** | **PC3** | **PC4** | **PC5** |
| --- | --- | --- | --- | --- | --- |
| Coarse sand fraction | 0.52 | 0.27 | 0.26 | -0.32 | -0.69 |
| Fine sand fraction | 0.28 | -0.64 | -0.66 | -0.10 | -0.24 |
| Total sand fraction | 0.55 | 0.05 | 0.03 | 0.83 | 0.07 |
| Silt | -0.45 | -0.48 | 0.41 | 0.35 | -0.53 |
| Clay | -0.38 | 0.53 | -0.57 | 0.28 | -0.42 |
| **Proportion of variance** | 0.65 | 0.23 | 0.11 | 0.00 | 0.00 |
| **Cumulative proportion of variance** | 0.65 | 0.88 | 1.00 | 1.00 | 1.00 |

Table S3: **Principal component analyses (PCA)** of the chemical proprieties: phosphorus (P), exchangeable bases (Na, K, Ca, and Mg), the sum of all exchangeable bases (SB), exchangeable aluminium (Al and H+Al), saturation index by aluminium (m), base saturation index (V), effective cation exchange capacity (t), and cation exchange capacity (T). Each principal component (PC) represents a proportion of variance of each variable. The table shows the total variance represented in each PC and the cumulative proportion of variance.

| **Variables** | **PC1** | **PC2** | **PC3** | **PC4** | **PC5** | **PC6** | **PC7** | **PC8** | **PC9** | **PC10** | **PC11** | **PC12** |
| --- | --- | --- | --- | --- | --- | --- | --- | --- | --- | --- | --- | --- |
| P | -0.26 | -0.20 | -0.53 | 0.69 | -0.34 | -0.06 | 0.07 | -0.10 | -0.01 | 0.00 | 0.00 | 0.00 |
| K | -0.29 | -0.29 | 0.04 | -0.05 | 0.39 | 0.22 | 0.78 | -0.15 | -0.02 | 0.00 | -0.01 | 0.01 |
| Na | -0.23 | -0.34 | -0.43 | -0.60 | -0.07 | -0.52 | -0.06 | -0.09 | 0.01 | -0.01 | 0.00 | 0.01 |
| Ca | -0.34 | 0.06 | 0.21 | -0.10 | -0.27 | 0.16 | -0.09 | -0.25 | -0.06 | -0.47 | 0.03 | 0.66 |
| Mg | -0.34 | 0.05 | 0.11 | 0.09 | 0.05 | -0.23 | 0.09 | 0.86 | -0.15 | -0.09 | 0.01 | 0.13 |
| Al | 0.15 | -0.48 | 0.50 | 0.32 | 0.16 | -0.51 | -0.10 | -0.18 | -0.12 | 0.01 | 0.20 | 0.11 |
| H_Al | 0.11 | -0.57 | -0.12 | 0.00 | 0.23 | 0.41 | -0.36 | 0.22 | 0.14 | -0.45 | -0.06 | -0.13 |
| Sb | -0.35 | 0.05 | 0.18 | -0.08 | -0.21 | 0.10 | -0.04 | -0.07 | -0.08 | -0.18 | 0.65 | -0.56 |
| t | -0.34 | -0.07 | 0.34 | 0.00 | -0.19 | -0.05 | -0.09 | -0.12 | -0.12 | -0.04 | -0.72 | -0.41 |
| T | -0.30 | -0.30 | 0.12 | -0.08 | -0.08 | 0.35 | -0.28 | 0.07 | -0.01 | 0.73 | 0.10 | 0.20 |
| V | -0.33 | 0.18 | 0.08 | 0.12 | 0.27 | -0.17 | -0.14 | -0.07 | 0.84 | 0.01 | 0.00 | 0.00 |
| m | 0.29 | -0.27 | 0.22 | -0.12 | -0.63 | 0.02 | 0.36 | 0.21 | 0.46 | 0.00 | 0.00 | 0.00 |
| **Proportion of variance** | 0.66 | 0.21 | 0.05 | 0.03 | 0.02 | 0.02 | 0.01 | 0.00 | 0.00 | 0.00 | 0.00 | 0.00 |
| **Cumulative proportion of variance** | 0.66 | 0.87 | 0.92 | 0.95 | 0.97 | 0.99 | 1.00 | 1.00 | 1.00 | 1.00 | 1.00 | 1.00 |

Table S4**: Model comparison** between the simple general linear model (GLM) and the stochastic partial differential equations (GLM + SPDE) using the integrated nested Laplace approximation (INLA) function. We modelled OTU richness and Shannon diversity index as dependent variables and soil characteristics as independent variables for eukaryotes, prokaryotes, litter, and soil. The model comparison is based on the deviance information criterion (DIC) and the Watanabe-Akaike information criterion (WAIC), where smaller values indicate better model fit. For all datasets – prokaryote (16S) and eukaryote (18S) soil and litter OTU diversity with soil characteristics – the best model was the one that included spatial correlation (values in bold).

| **Taxonomic group** | **Soil Horizon** | **Model** | **DIC** | **WAIC** |
| --- | --- | --- | --- | --- |
| **Richness** | | | | |
| Prokaryote | Soil | GLM | 401 | 432 |
|  |  | GLM + SPDE | **337** | **410** |
|  | Litter | GLM | 1172 | 1314 |
|  |  | GLM + SPDE | **698** | **914** |
| Eukaryote | Soil | GLM | 967 | 1081 |
|  |  | GLM + SPDE | **739** | **982** |
|  | Litter | GLM | 1384 | 1565 |
|  |  | GLM + SPDE | **1079** | **1425** |
| **Effective number of OTUs** | | | | |
| Prokaryote | Soil | GLM | 392 | 419 |
|  |  | GLM + SPDE | **327** | **392** |
|  | Litter | GLM | 1168 | 1307 |
|  |  | GLM + SPDE | **704** | **920** |
| Eukaryote | Soil | GLM | 970 | 1081 |
|  |  | GLM + SPDE | **733** | **977** |
|  | Litter | GLM | 1392 | 1572 |
|  |  | GLM + SPDE | **1070** | **1410** |

List of figures:


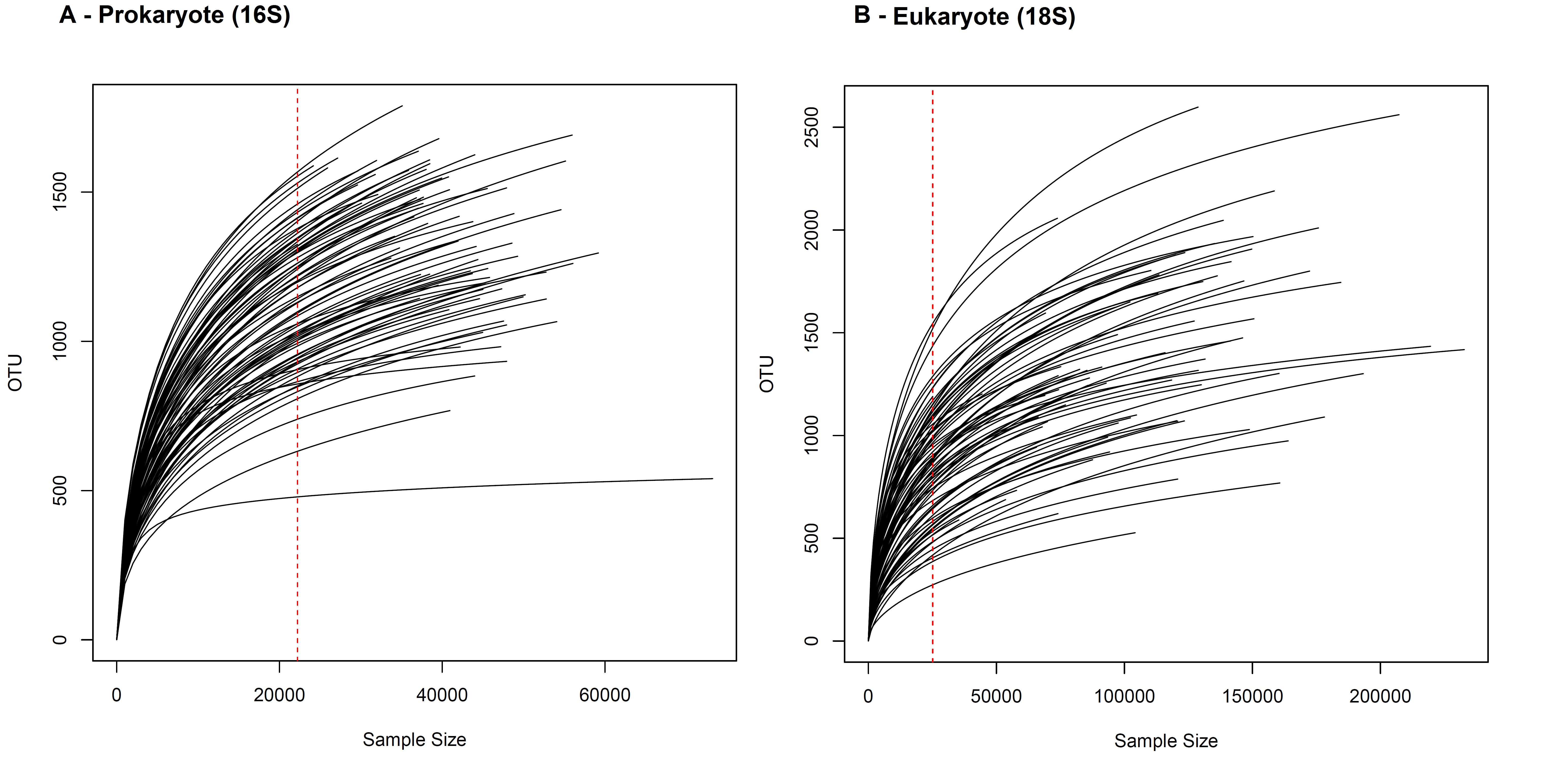


Figure S1**. Rarefaction curves.** Rarefaction by sample for the A) prokaryote dataset (16S) and B) the eukaryote (18S) dataset. The red line shows the smallest number of reads in a single sample, so all samples were downsized to this number of reads. The eukaryote data are more variable in read number than are the prokaryote data.

**
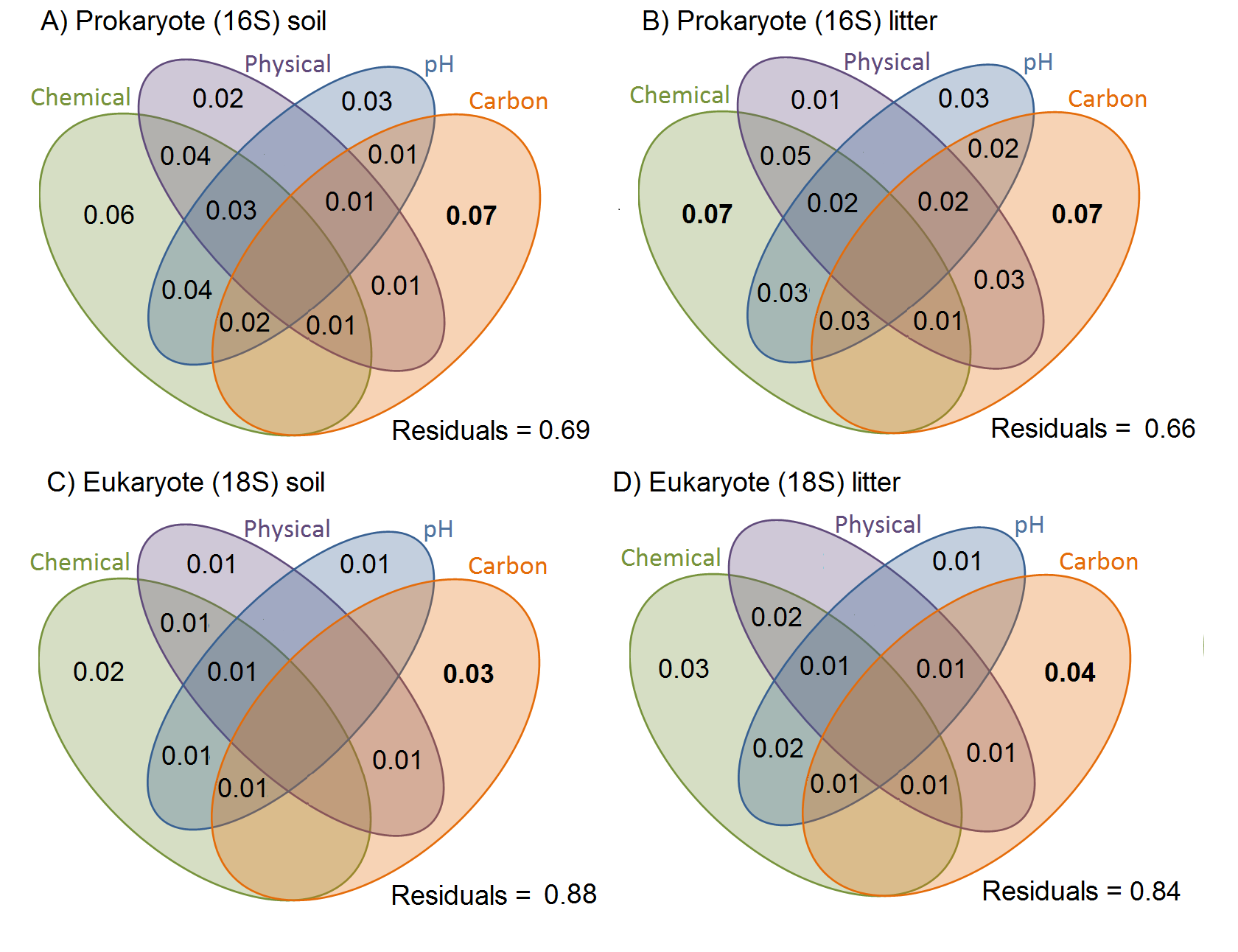
**Figure S2: **Variation in OTU community composition in the Amazonian soil samples explained by soil characteristics.** Results of the variance partition analysis based on Bray-Curtis dissimilarity distance-based analysis. Small but significant proportions of soil and litter communities vary with soil variables, and a small but significant proportion shows variation shared by soil variables. All values represent the proportion of variation explained by the factor or their interaction (Chemical + Physical, for instance). Chemical variables are shown in green (based on the first PCA of chemical variables, see Table S3 for details), physical variables in purple (based on first PCA axis of soil texture, see Table S2 for details), pH in blue, and carbon content in orange. The prokaryote communities are more structured by soil characteristics than are the eukaryote ones. Inside each taxonomic group, the litter communities are more structured by soil characteristics than are the soil communities.

**
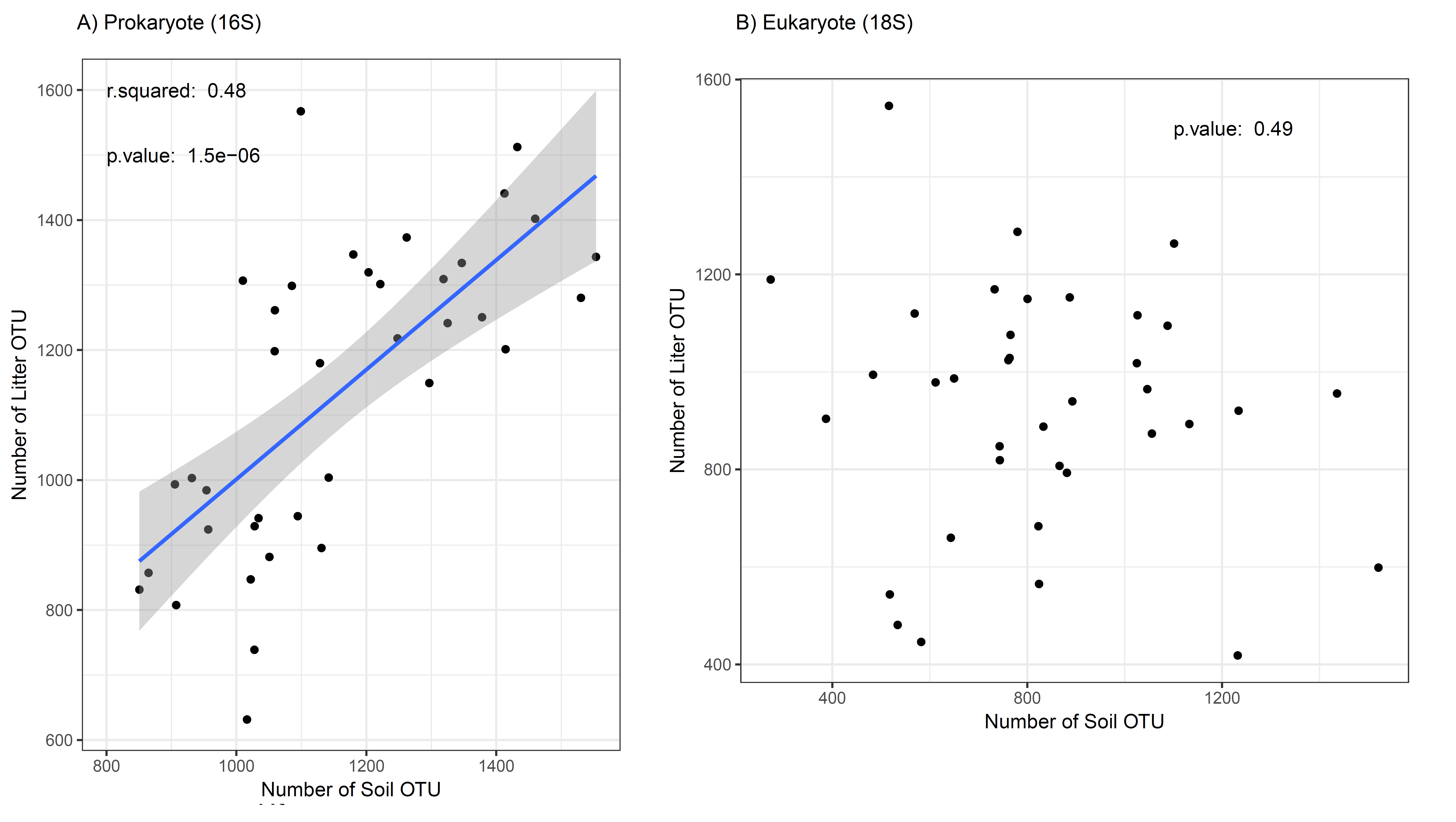
**

Figure S3: **Regression between prokaryote (16S) and eukaryote (18S) OTU richness.** The blue line shows a linear overall regression with standard error indicated by the shaded area for significant correlations. The regressions exclude the outlier “CXNCAMP3”, a move that improved the regression for prokaryotes in comparison to Figure 2**.**
